# Viable and necrotic tumor assessment from whole slide images of osteosarcoma using machine-learning and deep-learning models

**DOI:** 10.1371/journal.pone.0210706

**Published:** 2019-04-17

**Authors:** Harish Babu Arunachalam, Rashika Mishra, Ovidiu Daescu, Kevin Cederberg, Dinesh Rakheja, Anita Sengupta, David Leonard, Rami Hallac, Patrick Leavey

**Affiliations:** 1 The University of Texas at Dallas, Richardson, TX, United States of America; 2 The University of Texas Southwestern Medical Center, Dallas, TX, United States of America; 3 Children’s Medical Center, Dallas, TX, United States of America; University of California Riverside, UNITED STATES

## Abstract

Pathological estimation of tumor necrosis after chemotherapy is essential for patients with osteosarcoma. This study reports the first fully automated tool to assess viable and necrotic tumor in osteosarcoma, employing advances in histopathology digitization and automated learning. We selected 40 digitized whole slide images representing the heterogeneity of osteosarcoma and chemotherapy response. With the goal of labeling the diverse regions of the digitized tissue into viable tumor, necrotic tumor, and non-tumor, we trained 13 machine-learning models and selected the top performing one (a Support Vector Machine) based on reported accuracy. We also developed a deep-learning architecture and trained it on the same data set. We computed the receiver-operator characteristic for discrimination of non-tumor from tumor followed by conditional discrimination of necrotic from viable tumor and found our models performing exceptionally well. We then used the trained models to identify regions of interest on image-tiles generated from test whole slide images. The classification output is visualized as a tumor-prediction map, displaying the extent of viable and necrotic tumor in the slide image. Thus, we lay the foundation for a complete tumor assessment pipeline from original histology images to tumor-prediction map generation. The proposed pipeline can also be adopted for other types of tumor.

## Introduction

Examination of resected cancer specimens after delivery of chemotherapy allows pathologists to interpret the responsiveness of patient tumors and successfully influences patient outcomes by stratifying treatment administration to individual patient risk [1]. For high-grade osteosarcoma, tumor necrosis in response to pre-operative therapy has been a highly significant prognostic indicator for four decades [2]. Repeated international series emphasized the value of necrosis in predicting treatment outcome [3–5], and led to an extensive international study (EURAMOS-1) randomizing patients with high-grade resectable osteosarcoma to therapy based on tumor necrosis [6]. However, patient outcomes were not significantly improved by randomized therapy in the EURAMOS-1 trial [7, 8], leading some to challenge the validity of necrosis as a predictor in this disease [9]. It is clear from these data that limitations exist in the current approach to treating patients with osteosarcoma and while fundamentally this includes the need for novel therapies it also emphasizes the need for an effective indicator of treatment response to allow greater opportunity for personalized treatments. Non-invasive imaging with CT-PET [10, 11] and both diffusion-weighted and dynamic contrast-enhanced MRI [12, 13] might also be employed as biomarkers of therapy, but each are variably successful in predicting tumor response in high-grade osteosarcoma [9]. Limitations, therefore, in the predictive capacity of histological interpretation of necrosis and radiological interpretation of response suggest the opportunity to optimize both methods toward the development of an effective biomarker. These limitations include the fact that the histological estimation is typically available only after 10 weeks of pre-operative chemotherapy potentially too late to make meaningful adjustments in patient therapy. Limitations in histological assessment also include that the tissue processing protocol for resected specimens has not been significantly modified since it was initially described [2], and histological specimen preparation is time intensive. In fact processing requires manual handling and interpretation of as many as 50 histology slides per case yet it represents only a single plane of a large three-dimensional tumor. Radiological imaging for osteosarcoma typically does not include the use of enhanced MRI imaging sequences in routine care, and voxel level interrogation of large 3-dimensional tumors is not performed where the primary purpose is delineation of tumor margins for surgical planning.

Recent technology advances, in which histological tumor slides are converted to digital image datasets and in machine learning which can interrogate patterns in digital images of MRI and digitized histology may address some of these limitations. Specifically, for response evaluation in osteosarcoma, digital histopathology is made possible by scanning hematoxylin and eosin (H&E) stained microscopic slides [14] using commercially available scanning technology. The scanning converts a glass slide to a digital Whole Slide Image (WSI) that preserves image resolution up to 40X magnification. Although each WSI represents a very large digital file, interpretation is now feasible using image processing algorithms, while the advent of machine and deep-learning models make automated diagnostic systems a possibility.

The goals of this study are (1) to demonstrate the successful development of automated learning tools for the classification of osteosarcoma WSIs into regions of non-tumor, necrotic tumor, and viable tumor, and (2) to report areas of tumor and necrosis with high accuracy. It is critical in any machine learning operation that the automated learner be able to differentiate normal tissue from tumor tissue since there are elements of normal tissue in all resected cancer. The identified regions serve as a preparatory phase for calculating the percent of tumor necrosis in resected specimens. Ultimately our aim will be to combine the digital data from histology to co-registered planes of enhanced MRI sequences and using image pattern detection use MRI imaging to enhance the ability to individualize care for patients since 45% of all newly-diagnosed patients with osteosarcoma ultimately die of the disease. Advances in treatment is critically dependent on the development of novel methods [15], that can be assessed through automated tools, and our work demonstrates the first successful step toward such a tool.

The manuscript has been organized as follows. The section *Materials and Methods* breaks down the step by step construction of our image analysis pipeline for osteosarcoma. It gives a detailed account of the stages of data preparation and classification using machine-learning and deep-learning models. The *Results* section provides a comparison between feature categories, and analyzes the outcomes of machine-learning and deep-learning methods, using various performance evaluation metrics. The *Discussion* section summarizes the overall accomplishments in this study, establishes the context in which the results will be used, and concludes by sharing thoughts on future directions.

## Materials and methods

The various stages carried out in this study include the preparation of data, followed by learning and classification for machine-learning and deep-learning models. The results are then analyzed in a number of sub-steps on various performance evaluation metrics.

### Data preparation

Archival tumor samples, taken at the time of osteosarcoma resection from 50 patients treated between 1995 and 2015, were retrieved from the pathology archives at Children’s Medical Center, Dallas. From these samples, 942 histology glass slides (mean: 19 slides per patient; range: 4 to 51 slides per patient) were digitized into whole slide images (WSI).

#### Selecting image tiles for investigation

**Fig 1:**
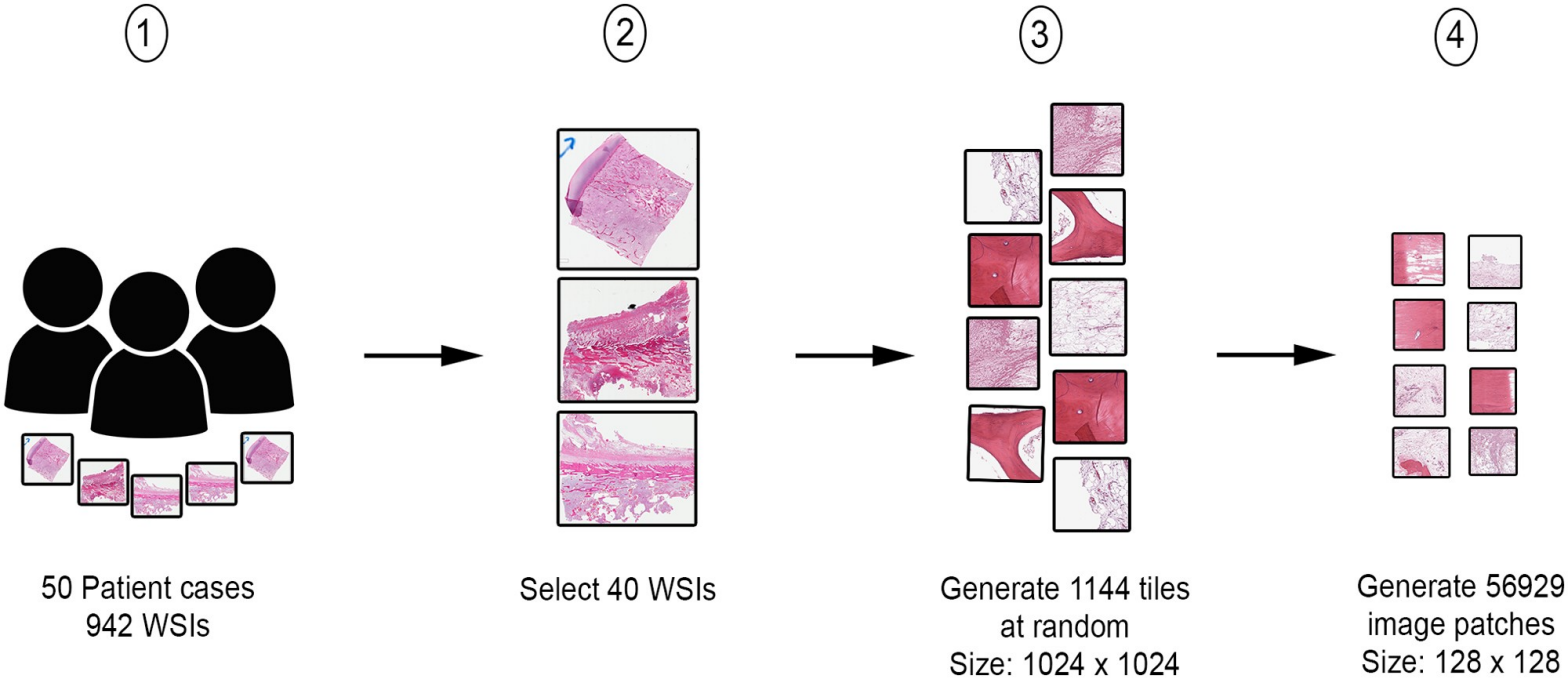
Data preparation process. Step 1 shows the assembly of patient archival samples of 50 cases, resulting in 942 WSIs. Step 2 involves the selection of 40 handpicked WSIs by a pathologist. In step 3, 1144 image tiles of size 1024x1024 are generated from WSIs identified in step 2. From each image tile in step 3, a number of image patches of size 128x128 are generated.

From the digitized images, 40 WSIs representing the heterogeneity of tumor and response characteristics under study, were manually selected by two pathologists. Thirty 1024 x 1024 pixels image tiles at 10X magnification factor were randomly selected from each WSI. Our choice of magnification was made following a discussion with the pathologists who confirmed that it is the best level to identify features separating the three classes of interest in a WSI image. A lower magnification for WSIs will loose the spatial information, while a higher magnification will include sub-cellular regions that are not relevant to the task and may negatively affect the segmentation process. From the resulting 1,200 image tiles, 1,144 were selected after removing irrelevant tiles such as those falling in non-tissue, ink-mark regions, and also blurry images. The randomization of tile-generation removes any bias in selection of dataset for the purposes of feature- generation and subsequent machine/deep-learning steps. Furthermore, from each image tile, a number of 128 × 128 size overlapping image patches were generated for input to the deep-learning model, which resulted in a total of 56,929 image patches. The generated 128 × 128 patches are manually annotated again with class labels based on the mask annotation by the pathologists on the tiles. A high-level view of the data preparation procedure is presented in Fig 1.

#### Ground truth for classification

The pathologists were provided with the generated image tiles, uniquely identified by a Tile Identification Number (TIN), for image annotation procedure. A tool was built in-house, specifically for the task of manual image-annotation by pathologists (S1 Fig). An appropriate class label was assigned, from the provided options, to each input tile. Color annotations were used for identifying the regions and were subsequently used for training the deep-learning model. All image tiles were divided between two pathologists for the annotation activity. Each image tile had a single annotation as any given tile was annotated by only one pathologist. The results were exported to a comma separated value (CSV) file, while the images with annotations were saved to separate image files. Each record of the CSV file contained a TIN and its corresponding classification result.

The three main regions used in all classification tasks are namely Viable tumor (VT), Non-tumor (NT) and Necrotic tumor (NEC). Expert annotation by two pathologists of 1,144 tiles resulted in classification as follows: 536 (47%) non-tumor tiles, 263 (23%) necrotic tumor tiles and 345 (30%) viable tumor tiles.

### Generating features for machine-learning

The image-tiles from the preceding stage were used as input for generating features. The images were processed through a number of steps, first by an application developed in-house [16] and then by CellProfiler [17]. The generated features were grouped into *Expert-guided* and *CellProfiler* generated categories.

#### Expert-guided features

**Fig 2:**
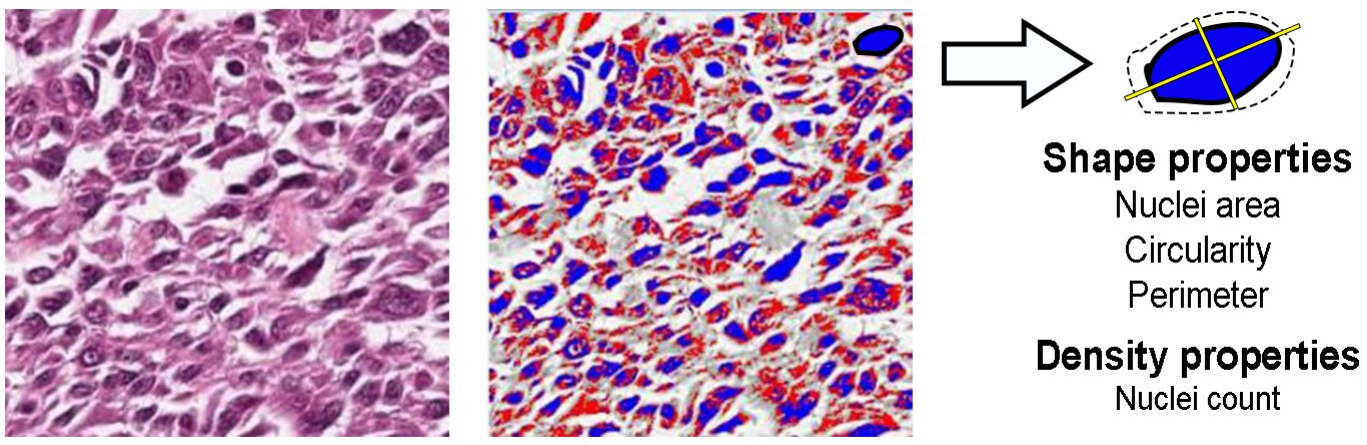
Expert-guided features. The result of Otsu thresholding method (right) for an image segment (left) is presented. The foreground pixels are marked in blue and the background pixels in red. The outcome was a number of shape and density properties for each image tile.

The properties that pathologists look for in WSIs are emulated as expert-guided features, using the steps described in [16]. Each image tile was subject to three levels of segmentation, namely color, shape, and density. The tiles were converted to LAB color space which divided the color channels into three dimensions L,a*,b*. A threshold of *δ* ≥ 64 (selected empirically), applied along a* dimension removed pixels that were part of the bone, red blood cells, and other false positive regions. Subsequently, a color and threshold segmentation method, based on Otsu thresholding [18], divided the stained images into foreground and background pixels, respectively marked as blue and red (Fig 2). A shape segmentation step identified nuclei and computed their shape properties such as circularity, area, perimeter, and center. Then the average number of nuclei in a 32x32 window was computed as a derived feature resulting in a total of eight features overall, generated from the dataset. Algorithm 1 details the steps involved in image segmentation and feature generation processes described above.

**Algorithm 1 Expert-guided feature generation method**

**Input:** A set of H&E stained images, Î = {*I*^1^, *I*^2^…*I*^*n*^}

**Output:** Set of features, $\hat{\text{X}}=\{X_{1},X_{2}\ldots X_{8}\}$

1: **for** image I^*k*^ ∈ Î **do**

2:  Convert I^*k*^ into LAB colorspace, and remove objects beyond threshold *δ* > 64 in a* dimension, resulting image denoted as I*.

3:  Segment I* using Otsu thresholding and divide pixels into red and blue color, resulting in image I^*O*^.

4:  From the previous step result, I^*O*^, run flood fill algorithm to compute clusters

5:  Count the number of clusters, number of blue and red pixels, area of each cluster, perimeter and circularity/ eccentricity, and add to $\hat{\text{X}}$

6: return $\hat{\text{X}}$

#### CellProfiler features

Texture features are important descriptors in any image processing task. Hence, to identify more image properties relevant to digital image analysis, we built an image-processing pipeline using CellProfiler [17]. The following steps detailed in Algorithm 2, were configured to process every image tile.

A color deconvolution [19] was performed on each image tile using *Unmix colors* module. The staining options for this step were set to hematoxylin and eosin stains. The result was a set of hematoxylin and eosin stained images in grayscale. The hematoxylin images were further processed through a texture segmentation filter using *Measure Texture* module. Haralick features [20] were calculated from the images by creating a Gray Level Co-occurrence Matrix (GLCM) which computed co-occurrence values based on values of adjacent pixels. Thirteen features were computed on the image by performing mathematical computation on the GLCM. Next, a color segmentation process by Otsu thresholding [18] used CellProfiler’s *Apply Threshold* module. The parameter was set to ‘Automatic’, and the threshold method was set to ‘Otsu’. The resulting image, after threshold segmentation, was subjected to a shape segmentation process using *Measure Primary Objects* module, which calculated the number of nuclei present in the image. A minimum diameter of 30 pixels and a maximum diameter of 120 pixels were configured as the size of objects to be identified. The primary objects identified from the previous step were used as a reference to identify secondary objects such as cells, cytoplasm, and portions of stroma using Measure Secondary Objects module. Count of objects, weighted variance between neighboring pixels, and sum of entropies between foreground and background were calculated as features from the above two steps. A density segmentation approach using *Measure Object Neighbors* module calculated the number of neighboring nuclei clusters in the identified primary and secondary objects from each tile. In total, a set of 53 features generated from CellProfiler were provided as input feature set for machine-learning models. The expert-guided feature set and the cellprofiler feature set are mutually exclusive. Only textural features with high information gain are retained from the cell profiler and overlapping features such as nuclei count are dropped.

**Algorithm 2 CellProfiler feature generation**

**Input:** A set of H&E stained images, Î = {*I*_1_, *I*_2_…*I*_*n*_}

**Output:** Set of features, $\hat{\text{F}}=\{f_{1},f_{2}\ldots f_{n}\}$

1: **for** image I^*k*^ ∈ Î **do**

2:  Perform color deconvolution using *Unmix Colors* module to separate the image into hematoxylin (I^*h*^) and eosin (I^*e*^) stain components respectively

3:  I^*h*^ grayscale image is analyzed for texture segmentation using *Measure Texture* module.

4:  The properties generated from Haralick texture method from the previous step are saved.

5:  I^*h*^ is processed again using Otsu thresholding, from the module *Apply Threshold*. The parameter is set to Automatic. The result is stored in an image I^*O*^.

6:  From the resulting I^*O*^, primary objects like nuclei and lymphocytes are identified, using *Measure Primary Objects* module. The minimum and maximum diameter values are set to 30 and 120 respectively. An *automatic* thresholding strategy is used.

7:  From identified primary objects, secondary objects are identified using *Identify Secondary Objects* module. The module calculates secondary objects such as cytoplasm, cell, portion of stroma etc.

8:  Perform density segmentation using *Measure Object Neighbors*, to calculate the number of objects clumped together

9:  Save the values generated in the previous step for each feature

10: return $\hat{\text{X}}$

### Machine-learning

Machine-learning models have been successfully used in various studies for accurate tumor prediction [21, 22]. The combined set of 61 features (53+8), generated in the previous step, were given as input for 13 machine-learners. We used three categories of models, namely Complex Decision Trees, Support Vector Machines, and Ensemble Learners, with an aim to identify the one with the best fit on the provided feature set.

#### Decision trees

Decision trees are one of the non-parametric approaches that divide input data points into axis-parallel hyper-rectangles and assign a response variable for each identified region [23]. For each feature X_*i*_ from our combined feature set, entropy is calculated using the following equation.

$$\begin{array}{c} H\left(X_{i}\right)=\sum_{j=1}^{\left|C\right|}-P\left(x_{j}\right)logP\left(x_{j}\right) \end{array}$$ (1)

where H(*X*_*i*_) is the entropy of feature *X*_*i*_, *C* is the set of values of target variable viable tumor, necrotic tumor and non-tumor, and P(*x*_*j*_) is the proportion of *X*_*i*_ belonging to a class under consideration. A feature *X*_*i*_’s relevance is found using the following equation.

$$\begin{array}{c} G\left(C,X_{i}\right)=H\left(C\right)-\{\sum_{x\in values(X_{i})}\frac{|C_{x}|}{\left|C\right|}H\left(X_{i}\right)\} \end{array}$$ (2)

where G(C,*X*_*i*_) is the information gain for class variable C and attribute X, values(*X*_*i*_) is the set of values that *X*_*i*_ takes, and *C*_*x*_ is the subset of C (i.e., one of the values of viable tumor, necrotic tumor, non-tumor) for which the value of X_*i*_ is x (i.e., *C*_*x*_ = c, X_*i*_(c) = x). The algorithm runs for many iterations, until convergence. In each iteration, the attribute with the highest gain value, G(C,*X*_*i*_), is selected and the input set is partitioned based on this attribute. The process of tree building runs until only one class remains for the input partition or until the termination conditions are satisfied.

#### Support vector machines

Support vector machine (SVM) [24] is a supervised learning method that maximizes a margin between the classification boundaries among different classes. Given a set of input features (*X*), their corresponding weights (*W*), and class labels (*Y*), SVM fits a hyper-plane between points by minimizing the following function (with slack variables).

$$\begin{array}{c} \frac{1}{2}∥W^{2}∥+C\sum_{i=1}^{n}\varepsilon_{i} \end{array}$$ (3)

subject to the inequality, *y*_*i*_(*w*^*T*^.*x*_*i*_ + *b*) − 1 + *ε*_*i*_ ≥ 0 (∀*i* ∈ *n*), where *n* represents the number of data samples, *C* is a parameter to control over-fitting, and *ε*_*i*_ is a slack variable. The above equation is transformed to include Lagrangian multipliers (*α*_*i*_) in order to handle inequalities better and introduce dot product between vectors. The objective of the transformed dual formulation becomes,

$$\begin{array}{c} maximize\;(\sum_{i=1}\alpha_{i}-\frac{1}{2}\sum_{i=1}\sum_{j=1}\alpha_{i}\alpha_{j}y_{i}y_{j}x_{i}^{T}x_{j}) \end{array}$$ (4)

subject to 0 ≤ *α*_*i*_ ≥ *C* and ∑_*i*_ *α*_*i*_.*y*_*i*_ = 0. Higher dimensional feature sets can be handled by configuring a kernel objective function (Eq 5) that can be linear, quadratic, cubic, Gaussian, polynomial etc. The resulting equation becomes

$$\begin{array}{c} maximize\;(\sum_{i=1}^{n}\alpha_{i}-\frac{1}{2}\sum_{i=1}\sum_{j=1}\alpha_{i}\alpha_{j}y_{i}y_{j}K\left(x_{i}^{T}x_{j}\right)) \end{array}$$ (5)

where $K({x_{i}}^{T}x_{j})$ is a kernel function that maps vectors *x*_*i*_ and *x*_*j*_ to a higher dimension, based on the type of kernel configured. Kernels such as polynomial, Gaussian, Laplacian, and exponential are used whenever the data is not linearly separable. In our experiments, among all configurations of SVM learners, the best performing model under the automatic operating point selection criteria was a kernel with a cubic polynomial function (degree 3) in the classification task, described below

$$\begin{array}{c} K\left(x_{i}^{T},x_{j}\right)={\left(x_{i}^{T}.x_{j}+1\right)}^{d} \end{array}$$ (6)

In the above equation, *d* represents the degree of the polynomial and the kernel used a d = 3 setting for our hierarchical classification task.

#### Ensemble learning

Bagging and Boosting are two ensemble learning methods that can reduce variance and bias, respectively [25]. Bagging is a bootstrapping method that uses repeated random sampling with replacement of data, increasing the data samples, hence reducing variance. Boosting selects a subset of data and trains on it against weak classifiers in a series of iterations. The output of one iteration serves as the input for next iteration. The weights of points misclassified in an iteration are improved for use in the next iteration, using a weight update function. In the experimental setup considered, decision trees were used as the base classifiers for learning and classification in the bagging and boosting models.

#### Parameter configuration for machine-learning models

We used four different complex tree models by varying the number of splits between 30 and 100. We changed the splitting criteria between Gini’s diversity index and maximum deviance reduction. We applied six configurations of support vector machines from MATLAB’s classification learner application settings, the main difference being the kernel function, which alternated between quadratic, cubic, and Gaussian kernels. We used one-vs-one and one-vs-all approaches for handling multi-class variables. For ensemble learners, we used three models of bagged and boosted trees in our trials. The core algorithm was decision trees over which we used the AdaBoost [26] algorithm for training. The parameters were selected by evaluating the models with a 5 fold cross-validation set. It follows that our analysis of machine-learning models performance across the main classes was quite extensive, allowing us to select the best performer overall. All experiments were carried out in MatLab’s classification learner package [27].

### Deep-learning

In recent years, deep-learning using neural networks has proved to be very successful in image classification. After ResNet [28], a residual block neural network won the Large Scale Visual Recognition Challenge, the focus on deep-learning for solving image classification tasks has increased. It was not long before deep-learning was used to solve medical imaging problems. Ciresan et al. [29] were among the first to apply Convolutional Neural Network (CNN) to mitosis counting for primary breast cancer grading. Histopathology image processing soon caught up with deep-learning when Litgens [30] et al. applied a CNN to breast cancer detection. Other implementations of deep neural networks include a fast scanning deep CNN for breast cancer detection [31]. The above works bear testament to the fact that deep-learning is a reliable method of image classification. To leverage the power of this technique, we built a deep-learning CNN model [32] as an extension of AlexNet [33] and LeNet [34]. The model includes three convolution layers, three sub-sampling layers, and two fully connected multi-layer perceptrons. The fully trained network can classify all the three tumor subclasses, viable, necrosis, and non-tumor (VT, NEC, and NT), with high accuracy. The model requires only annotated image data, from which it can learn abstract features as described in Rashika et al. [32].

#### Configuring the CNN

**Fig 3:**
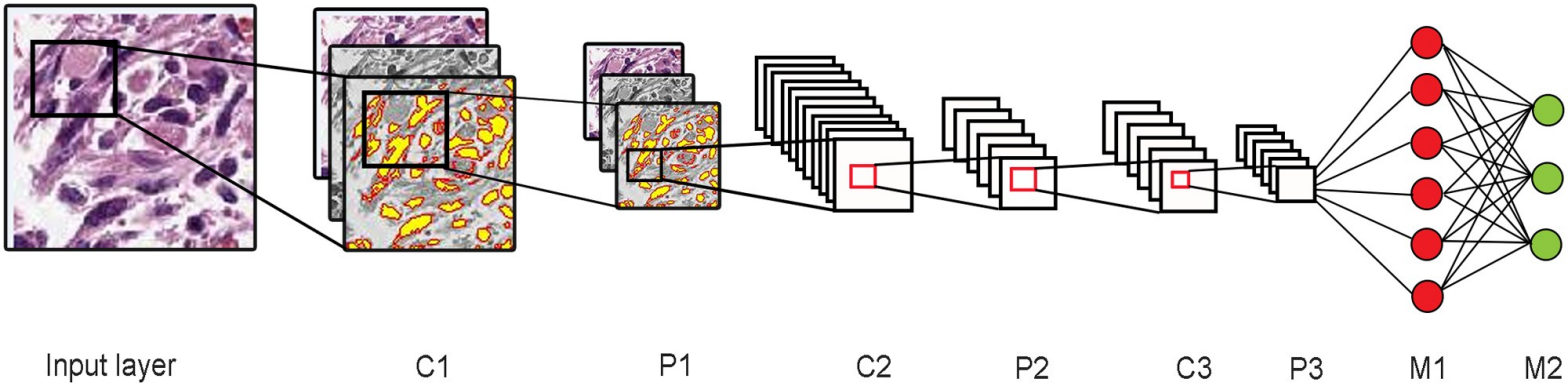
Deep-learning architecture. Image shows the deep-learning convolutional neural network architecture used in this study.

The architecture for the CNN based neural network consists of three convolution layers, three sub-sampling layers, and two fully connected multi-layer perceptrons (Fig 3). There is an input layer that accepts raw image pixel values of RGB. Each input is a sub-tile patch of size 128 x 128 pixels. A convolution layer computes the dot product of raw input values that are connected to local neurons and their corresponding weights. Max pooling layer follows the convolution layer and down-samples the volume along the spatial dimensions to 62x62x4. The Convolution and max-pooling layers are alternated three times in our architecture, a choice made through extensive experimentation. The first two pairs of layers have a filter size of 5x5 and a down-sampling scale of 2, while the last layer pair has a filter size of 3x3 and a down-sampling size of 2. There are two fully connected multi-layer perceptrons (MLP) that follow the three-layer pairs, containing a number of hidden layers. A logistic regression is used as the activation function. The second layer of MLP has three output neurons that produce a probability value for each of the three output classes. The network is trained via stochastic gradient descent with nesterov momentum (gamma value set to 0.9) where all weights are initialized with small random numbers from a gaussian distribution of *N*(0, 0.01). The model hyper-parameters are set using the “babysitting approach” in which they are manually tuned through various runs of the deep learning model. Based on the convergence of gradient descent from the various runs, the model is run for 20 epochs with learning rate and batch size set to 10^−3^ and 100 respectively.

#### Data preprocessing

All image patches were processed to remove patches with insignificant information by converting the image to binary and calculating its Euler value. The Euler value is the total number of objects in the image minus the total number of holes in those objects. It identifies images that contain mostly background (white) pixels. An Euler threshold of e ≥ -3 (selected through experimentation) was used to identify significant image patches for training. This value was reached by testing euler threshold values in (−15 < *e* < 15) range on a validation set of 1000 patches containing 300 examples with only background. The remaining patches were then normalized to alleviate the effect of illumination and contrast conditions while scanning the histology slides. This was done by transforming the RGB channel input into Lab color space and normalizing the L (lightness) parameter of the L*a*b color space.

#### Data augmentation

Data augmentation is the method of adding more data samples pooled from the already available data through transformation methods that preserve or alter properties of data. This has particular use in deep neural network learning since every model requires a large amount of data to train. It also plays a major role in avoiding over-fitting of data in our architecture. We considered two augmentation techniques. The first was a transformation operation through rotation of the image by 90°,180°and 270°, in addition to flipping/mirroring the image along vertical and horizontal axes. Each image snip resulted in eight transformations that are added to the dataset. The second technique applied a principal component analysis on each pixel along each RGB channel. If for each image pixel, *I*_*xy*_ = {*p*_*r*_, *p*_*g*_, *p*_*b*_} (x and y are the pixel locations), *p*_*r*_, *p*_*g*_, *p*_*b*_ are red, green and blue channel values respectively, then the principal component, *φ* is calculated as

$$\begin{array}{c} \varphi \left(x,y\right)\;=\;\left[q_{1}\;q_{2}\;q_{3}\right].{\left[\lambda_{1}\alpha_{1}\;\lambda_{2}\alpha_{2}\;\lambda_{3}\alpha_{3}\right]}^{T} \end{array}$$ (7)

where *q*_*i*_ and λ_*i*_ are the Eigen-vectors and Eigen-values of 3x3 co-variance matrix of RGB pixel values, and *α*_*i*_ is a random value drawn from a Gaussian distribution N(1,0.1). The image matrix is of size 128 × 128 × 3. Let the number of training images before augmentation be *n* = 56, 929. Then the covariance matrix is calculated on an input matrix of size *n* × (128 × 128) × 3. We then calculate the eigen vectors and eigen values of the covariance matrix. We sort the eigen values in descending order and select the corresponding eigen vectors to the sorted eigen values as our features. We then find the principal components of the input data through multiplication and add a value *α* to each pixel of the image using Eq 7. This results in output images with perturbed principal color while still maintaining the original features.

## Results

The ultimate goal of this study is to demonstrate the ability of automated learners to identify viable-tumor and necrotic-tumor regions. The experimental results are analyzed in a sequence of steps effectively as follows.

1. We assess the information content of the generated features and compare expert-guided and CellProfiler features.
2. We present the results of 13 machine-learning models that led to selecting the one with the best fit for the provided features.
3. We evaluate the performances of best machine-learning model and deep-learner based on their ability to discriminate tumor from non-tumor and then viable from necrotic tumor.
4. We generate image-tiles from a WSI and use the learners to predict the classes. The classification outcome is then mapped to an eye-fit view of the WSI to create a tumor-prediction map.

### Performance evaluation metrics

The results shared in the succeeding paragraphs were reported based on the following standard metrics of performance evaluation.

#### K-fold cross-validation

Entire data is divided into K sets, each containing *N/K* samples, where *N* is the total number of data points. The training and validation are repeated for *K* iterations. In every trial K-1 sets serve as training data and one set serves as test data. The error reported is the average error over K iterations, and represents final classifier performance.

The accuracy and error reported give a good approximation of a classifier’s decision making ability. K-fold cross validation is a commonly accepted way of measuring the performance of a classification model.

#### Holdout cross-validation

The data is divided into training and test set based on a percentage split decided beforehand. The learners are trained on the training set and evaluated on the test set. In this study, we employ an 80-20 split for training and testing sets. In order to reduce bias, we use a stratification approach, which preserves the original distribution of class samples in training and test sets.

#### Accuracy computation

The machine-learning models are compared on their ability to accurately identify the three classes. The overall accuracy (O) is calculated as

$$\begin{array}{c} O=\frac{T_{NT}+T_{NEC}+T_{VT}}{N_{NT}+N_{NEC}+N_{VT}} \end{array}$$ (8)

where *T*_*NT*_, *T*_*NEC*_ and *T*_*VT*_ are the correctly classified samples in each class and *N*_*NT*_, *N*_*NEC*_ and *N*_*VT*_ are the total number of samples in specific classes.

The class specific accuracy ($C_{c}$) is calculated by the following equation.

$$\begin{array}{c} C_{c}=\frac{T_{c}}{N_{c}} \end{array}$$ (9)

where *c* represents the class variables (NT, NEC, VT).

### Analyzing feature importance

Expert guidance by two pathologists, differentiating histological features of non-tumor, viable tumor, and necrotic tumor, resulted in 8 programmable expert-guided features. An automated image processing tool, CellProfiler [17], was used to generate an extensive set of features, out of which we have retained the top 53. All these selected features were textural features. Features from both these sources informed the machine-learning models. We performed a comparative analysis of expert-guided and CellProfiler features by studying their information gain values. Information gain of a particular feature is the difference in information available for predicting the target class with or without that feature, with information reflecting how the predicted probabilities are distributed among target classes. Information gains of individual expert-guided and top CellProfiler features are summarized in Table 1. Expert-guided features had lower gains by themselves than features generated by CellProfiler. For example, the highest gain value for expert-guided features was 0.2014, for the feature ‘Total clusters’ in the image, while for CellProfiler it was 1.8406, for the feature ‘Texture Gabor’.

**Table 1:** Comparison of information gain ratio of top features from expert-guided and CellProfiler categories.

Feature	IG	Description
**Expert-guided features**		
Total clusters	(0.2014)	The number of nuclei clusters computed in an image tile
Average clusters	(0.2014)	The average number of nuclei clusters computed in a 32x32 window
Red count	(0.1572)	The number of background pixels in an image tile
Red percentage	(0.1572)	The percentage of red pixels among foreground and background pixels
Blue count	(0.1523)	The number of foreground pixels in an image tile
Blue percentage	(0.1523)	The percentage of blue pixels among foreground and background pixels
Area	(0.0874)	The average area of identified nuclei clusters in an image tile
Circularity	(0.0425)	The average circularity of identified nuclei clusters in an image tile
**CellProfiler features**		
Texture Gabor	(1.8406)	The Gabor feature measures striped texture in an object
Texture sum Entropy	(1.8406)	The sum of entropy of all pixel values in GLCM
Texture sum variance	(1.8406)	The variance of entropy values in GLCM
Texture variance	(1.8406)	The variance of GLCM elements in the matrix
Texture sum average	(1.8406)	Sum of product of entries with probability of co-occurrence of values in GLCM
Texture correlation	(1.8406)	Gray level linear independence between pixels
Texture angular second moment	(1.8406)	Measure of homogeneity in an image

IG—Information Gain

GLCM—Gray Level Co-occurrence Matrix

To develop insight into the relative contributions of the expert-guided and CellProfiler features, we trained the top configurations of the machine-learners using only the expert-guided features or CellProfiler features. The overall accuracy achieved by these learners is reported in Table 2. Although features from CellProfiler alone achieve higher overall accuracy than expert-guided features alone, it is evident that learning from both sets of features is better than learning from one or the other (Table 2). Removal of the expert-guided features decreases the final accuracy of the model by almost 7%. It can be argued that the expert-guided features encodes subtle image properties that the pathologists use to distinguish between different types of tissues.

**Table 2:** Comparison of accuracy of best machine-learners on various feature categories.

	Complex trees (CT)	Support Vector Machine (SVM)	Ensemble Learners (ENS)
*Total accuracy using only expert-guided or CellProfiler features*			
Expert-guided	65.7	70.5	68.0
CellProfiler	77.4	82.7	80.2
*Class specific and total accuracy using combination of expert-guided and CellProfiler features*			
Viable tumor (VT)	77	91	88
Necrotic tumor (NEC)	75	87	80
Non-tumor (NT)	87	91	90
Overall accuracy (O)	80.9	89.9	86.8

### Reporting overall and class-specific accuracies

The overall and class-specific accuracy of the machine-learners from combined features is reported in Table 2. Accuracy was estimated using 5-fold cross-validation of the data set. Overall accuracy ranged from 80.2% to 89.9%. SVMs achieved the highest overall accuracy (range 83.0%–89.9%) followed by ensemble learners (81.1%–86.8%) and complex trees (80.2%-80.9%). For any particular learner, class-specific accuracy was greatest for non-tumor, followed by viable, and necrotic tumor.

The overall and class-specific accuracy of the deep-learner was also evaluated on the same data set (Table 3). The deep-learner achieved overall accuracy of 93.3% and class-specific accuracies of 91.9% for non-tumor, 95.3% for viable tumor, and 92.7% for necrotic tumor.

**Table 3:** Deep-learning classification results.

Tumor sub-type	No. of annotated patches (128x128)			Patch accuracy in %	Tile accuracy in %
	Total	Training	Testing		
VT	15231	10662	4569	95.3	92.6
NEC	14827	10379	4448	92.7	91.5
NT	20806	14564	6242	91.9	89.5
Overall accuracy (O)				93.3	91.2

### Hierarchical receiver-operator characteristics

The 1,144 selected image tiles were randomly divided 80/20 into training and testing sets for hierarchical evaluation. The training set of 914 tiles included 428 (47%) non-tumor tiles, 210 (23%) necrotic tumor tiles and 276 (30%) viable tumor tiles. The testing set of 230 tiles included 108 (47%) non-tumor tiles, 53 (23%) necrotic tumor tiles and 69 (30%) viable tumor tiles. The above information is presented in Table 4.

**Table 4:** Table of ground truth for comparative evaluation. The representation of image tiles of all class labels in training and test sets is presented below.

Classification label	No. of image tiles		Per class distribution of tiles (%)
	Training set (80%)	Test set (20%)	
NT	428	108	47%
NEC	210	53	23%
VT	276	69	30%

**Fig 4:**
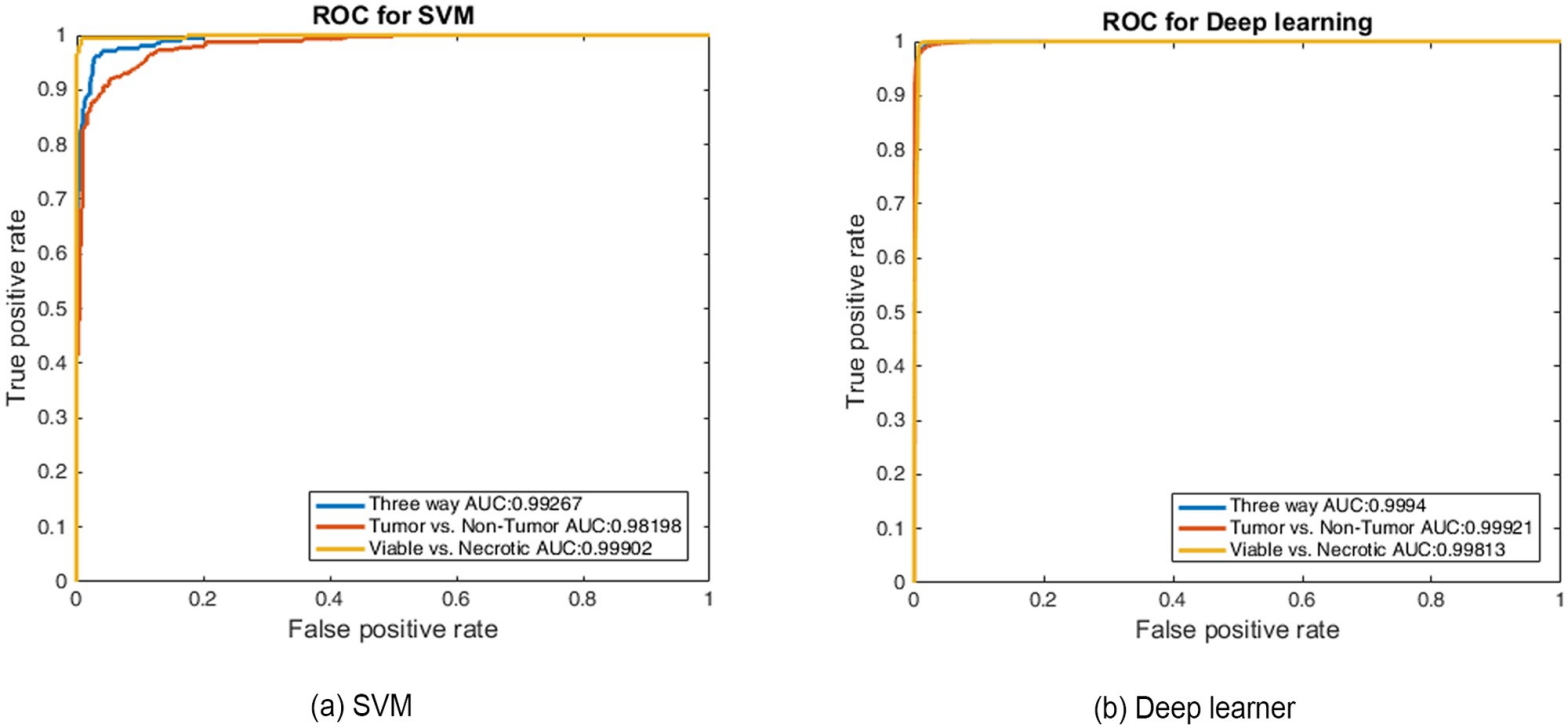
Area under the curve (AUC). Receiver operating characteristic curves presented for (a) SVM3 and (b) deep-learner shows AUC values ≥ 0.98 for both the learning models.

**Fig 5:**
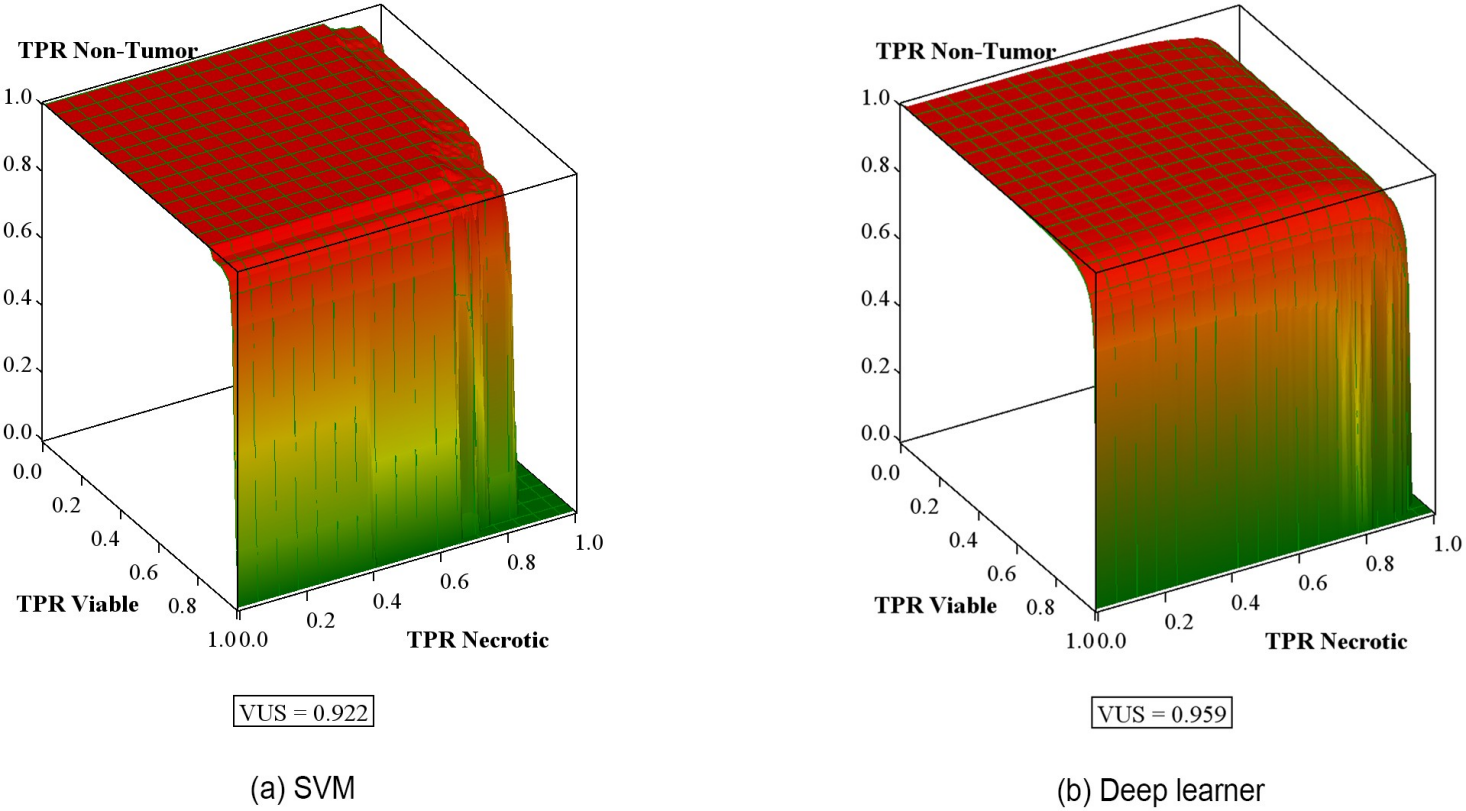
Volume under the surface. Receiver-operating characteristic surface for discrimination of non-tumor from tumor followed by conditional discrimination of necrotic from viable tumor with volume under surface (VUS) for (a) SVM3, (b) deep-learner. True positive rates (TPR) are within-class fractions of correctly classified image tiles/patches.

After retraining, the performance of SVM3 and the deep-learner in discrimination of non-tumor from tumor, followed by conditional discrimination of necrotic from viable tumor, was further assessed on the test data set by constructing the corresponding hierarchical receiver-operator characteristics. Receiver operating characteristic (ROC) curve analysis is widely used in biomedical research to assess the performance of diagnostic tests. The ROC curve depicts the quality of a diagnostic marker in a two-class classification problem. It illustrates the trade-off between sensitivity and specificity as a cut-off point for decision making. Area Under the ROC Curve (AUC) is the most widely used index for the quantification of the performance of a diagnostic marker in the two-class setting. Fig 4 shows the ROC curves for Tumor vs. Non-Tumor for the SVM classifier and deep-learner. For a 3-class setting, the Volume Under the Surface (VUS) was proposed as an index for the assessment of the diagnostic accuracy of the marker under consideration [35]. These surfaces are shown in Fig 5.

The AUC values for tumor and non-tumor graph are ≥ 0.98. This high value states that the SVM and deep-learner are able to separate tumor from non-tumor regions with a high confidence. But AUC values in a 3-class setting do not show the complete picture for positive class vs. negative class. Hence we compute VUS values as explained in the following section. The VUS value for SVM and the deep-learner were 0.922 and 0.959, respectively, which indicate both learners performed exceptionally well (VUS = 1 for perfectly informed classification; VUS = 0.167 for uninformed classification) in their respective classification tasks. VUS values are more robust towards small changes in a 3-class setting as compared to surface area.

#### Visualization of output

**Fig 6:**
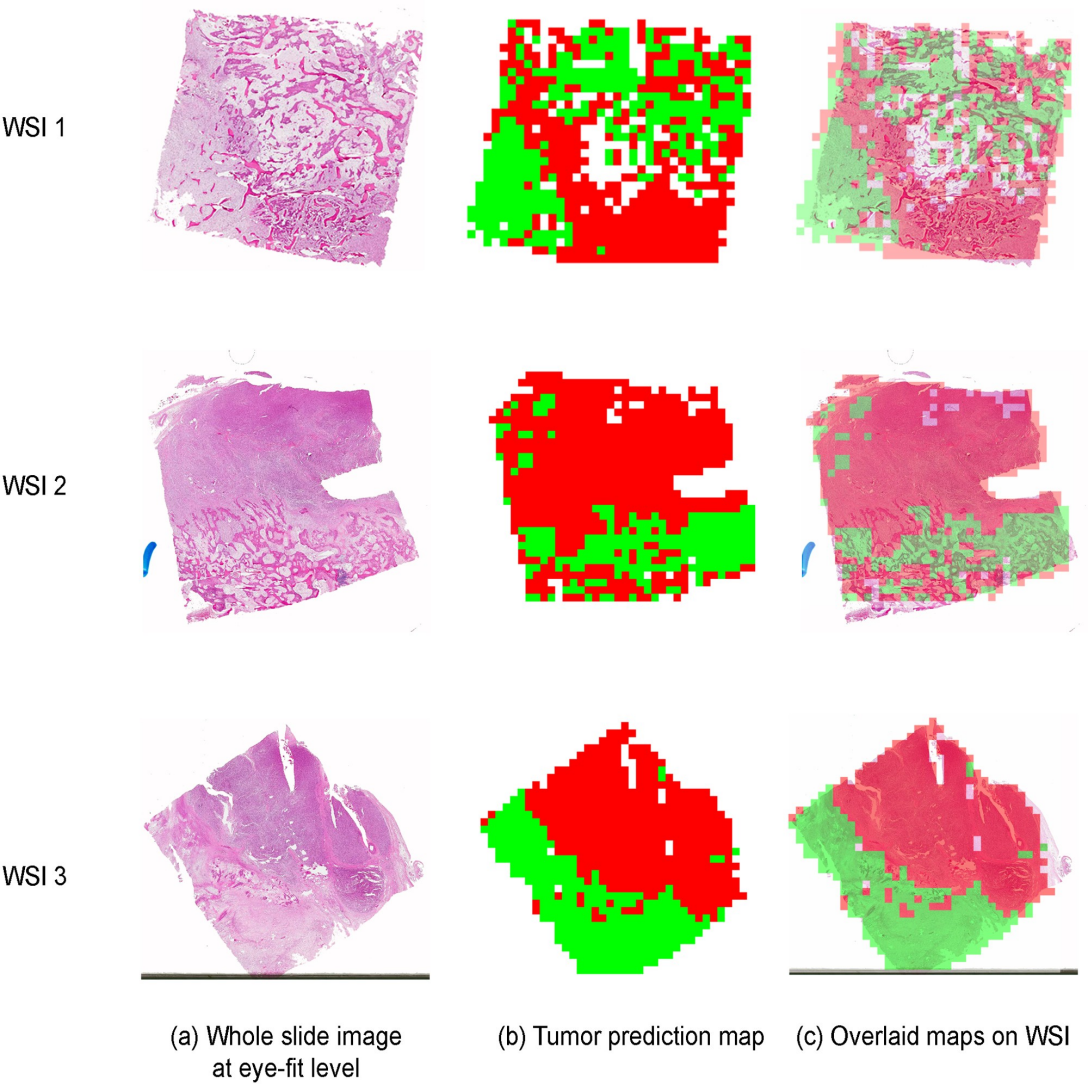
Tumor-prediction map. The figure shows the output for 3 WSIs. Image-tiles generated from a WSI (a) were provided as input to the learning models. The output of the classification task was used to create a tumor-prediction map (b), red signifying VT, green, NEC and blue, NT. The final images shows the overlaid tumor map on the WSI (c).

The trained models were used to analyze whole slide histology images and segment the WSI into different regions. From each WSI, 1000 to 1800 image-tiles of size 1024 X 1024 were generated. These tiles were then classified by the models and the results were mapped back to the original WSI at eye-fit level to generate tumor-prediction map. The map displays viable tumor in red, necrotic tumor in green and non-tumor regions in blue. The mapping step and the final tumor-prediction map are shown in Fig 6.

## Discussion

Osteosarcoma is a highly heterogeneous tumor and histological estimation of tumor response after ten weeks of therapy is time intensive and limited in its value. In this paper we present the first automated, accurate classification of WSIs (representing post-therapy resection specimens with osteosarcoma,) into viable tumor, non-tumor, and necrotic tumor. This is also the first study in osteosarcoma where necrotic tumor is automatically assessed from WSIs. We have generated digital histology properties based on nuclei features, spatial features, distance-based features and textural features from the input image tiles, for three categories of tissue within histology images, viable tumor / necrotic tumor/ areas of non-tumor. We have configured and tested traditional machine-learning models by combining expert identified features with CellProfiler features as a single input set, and selected the best performing model, an SVM. In parallel, we also built a deep-learning neural network based on the Alexnet [33] and LeNet architectures [34], which used a gradient-based learning with back-propagation algorithm. On our test data set, the accuracies and VUS reported by both traditional (selected SVM) and deep-learning models are comparable and very significant. The fact that both models, based on completely different approaches for tile classification, achieve similar results, is in itself a validation of the strength of this work.

Machine-learning has been implemented in many spheres of medicine with varying acceptance [36, 37]. Within the field of histopathology for cancer, automated learning algorithms have been successfully evaluated in renal clear cell carcinoma, glioma, gastric carcinoma, breast cancer, prostate cancer, and non-small cell lung cancer [21, 22, 38–41]. Specifically, deep-learning convolutional neural networks (CNN) accurately identified micro-metastasis in lymph node biopsies and prostate biopsies [30]. machine-learning tools identified histopathological features in non-small cell and squamous cell lung cancer and using these features, investigators were able to separate prognostic subgroups [22, 41], while CNN identified various subtypes of glioma with 90-96% accuracy [38]. Each of these reports utilized the ability of automated learning algorithms to recognize and segment cellular and environmental details. Identification of tissue necrosis, as noted in this report, represents a different level of complexity. Data describing deep-learning analysis of gastric carcinoma report 81% accuracy in detecting tumor necrosis [39] while data in glioblastoma both highlights the evolutionary features of necrosis and confirms the suitability of deep neural networks in histopathology image interpretation [42].

Limitations of this work include that the pathological evaluation of tissue samples is prone to inter-observer variability [43] and some of the features we used as input to the automated machine-learner depended on pathologist identified features. To adjust for this we identified textural features using an image analysis platform and in parallel developed a neural network that functions independently of expert-guided or textural feature input.

To our knowledge, the data presented in this report represent the first description using automated learner tools in the histological classification in high-grade osteosarcoma. We have optimized a pipeline for this interpretation, and highlighted multiple novel achievements toward this end. We developed an annotation tool for expert pathology review of osteosarcoma tiles for classification purpose, which allowed efficient and expert review of 1,144 image tiles, while simultaneously generating data for our machine-learning and deep-learning algorithms. We have optimized an image-processing platform using the Cellprofiler software, and identified 53 features in addition to those distilled and extrapolated from expert pathology examination. The high VUS value for both learners prove the robustness of the learned models on the blind test dataset.

As a final validation step, tumor-prediction maps were generated to display the classified tumor regions. These maps can be used to calculate the percentage of tumor necrosis in a patient and also visualize the extent of osteosarcoma over the whole slide image.

The work presented in this paper lays the complete framework for histopathological image analysis in osteosarcoma. We have demonstrated that the provided framework perform exceptionally well in the classification task. The same framework can also be adopted for other types of tumor.

## Supporting information

S1 Fig Screen-shot of the annotation software. The screen-shot shows an example region annotation regions on a histology image.
(TIF)
