# Supplementary figures and images for: Viable and necrotic tumor assessment from whole slide images of osteosarcoma using machine-learning and deep-learning models
Source: PLoS One. 2019 Apr 17;14(4):e0210706. doi: 10.1371/journal.pone.0210706 (PMC6469748; doi:10.1371/journal.pone.0210706)

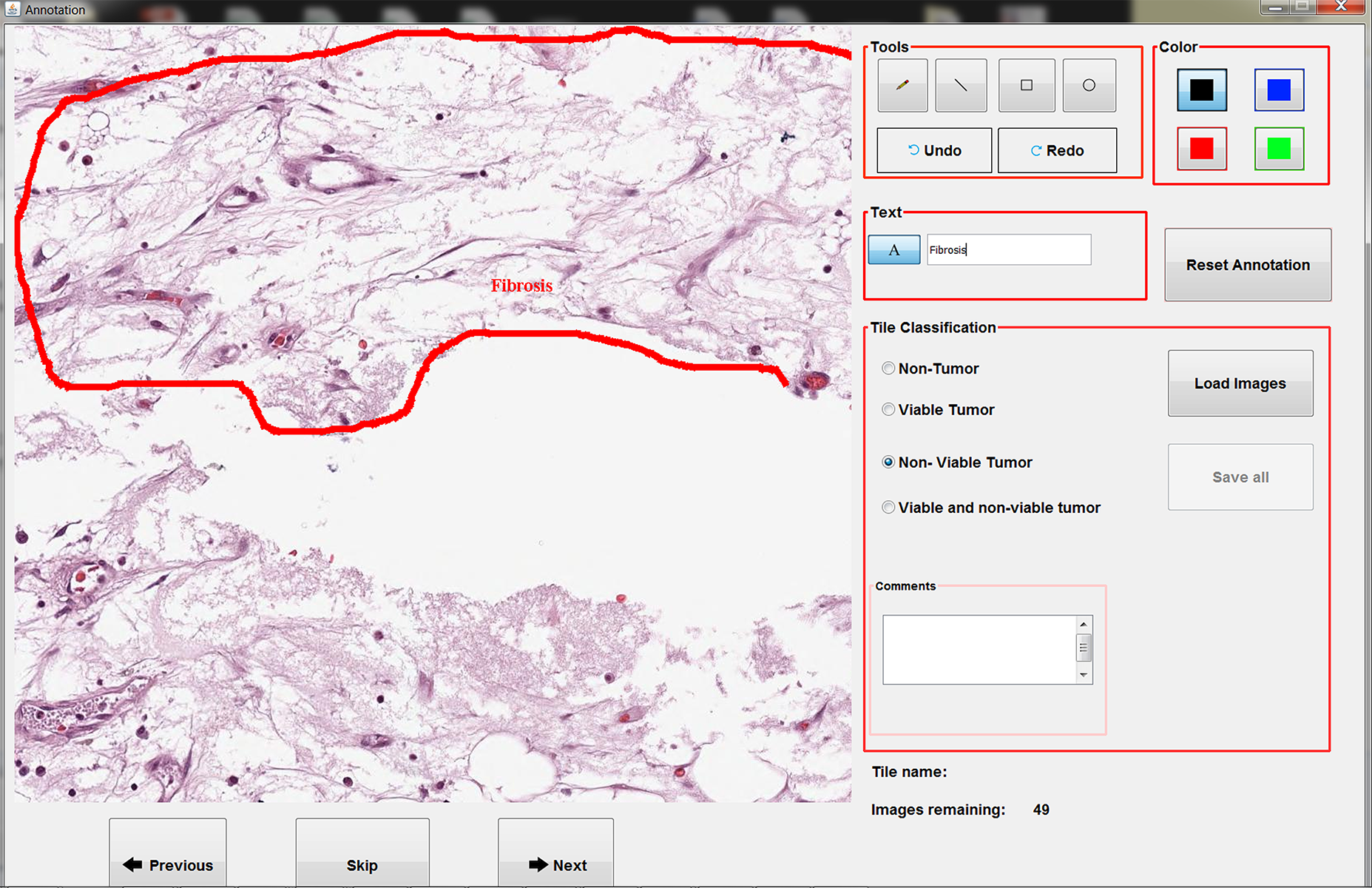

Supplement: S1 Fig — The screen-shot shows an example region annotation regions on a histology image. (TIF) [file pone.0210706.s001.tif]
